# Consequences of Warming and Resource Quality on the Stoichiometry and Nutrient Cycling of a Stream Shredder

**DOI:** 10.1371/journal.pone.0118520

**Published:** 2015-03-04

**Authors:** Esther Mas-Martí, Anna M. Romaní, Isabel Muñoz

**Affiliations:** 1 Departament d’Ecologia, Facultat de Biologia, Universitat de Barcelona (UB), Diagonal 643, 08028, Barcelona, Catalonia, Spain; 2 Institute of Aquatic Ecology, University of Girona, Campus de Montilivi, 17071, Girona, Catalonia, Spain; University of Yamanashi, JAPAN

## Abstract

As a result of climate change, streams are warming and their runoff has been decreasing in most temperate areas. These changes can affect consumers directly by increasing their metabolic rates and modifying their physiology and indirectly by changing the quality of the resources on which organisms depend. In this study, a common stream detritivore (*Echinogammarus* *berilloni* Catta) was reared at two temperatures (15 and 20°C) and fed *Populus nigra* L. leaves that had been conditioned either in an intermittent or permanent reach to evaluate the effects of resource quality and increased temperatures on detritivore performance, stoichiometry and nutrient cycling. The lower quality (i.e., lower protein, soluble carbohydrates and higher C:P and N:P ratios) of leaves conditioned in pools resulted in compensatory feeding and lower nutrient retention capacity by *E*. *berilloni*. This effect was especially marked for phosphorus, which was unexpected based on predictions of ecological stoichiometry. When individuals were fed pool-conditioned leaves at warmer temperatures, their growth rates were higher, but consumers exhibited less efficient assimilation and higher mortality. Furthermore, the shifts to lower C:P ratios and higher lipid concentrations in shredder body tissues suggest that structural molecules such as phospholipids are preserved over other energetic C-rich macromolecules such as carbohydrates. These effects on consumer physiology and metabolism were further translated into feces and excreta nutrient ratios. Overall, our results show that the effects of reduced leaf quality on detritivore nutrient retention were more severe at higher temperatures because the shredders were not able to offset their increased metabolism with increased consumption or more efficient digestion when fed pool-conditioned leaves. Consequently, the synergistic effects of impaired food quality and increased temperatures might not only affect the physiology and survival of detritivores but also extend to other trophic compartments through detritivore-mediated nutrient cycling.

## Introduction

Climate warming is unequivocal, and even greater increases in temperature and changes in precipitation are predicted for this century [1]. Consequently, rivers and streams in many regions are predicted to warm and experience changes in their runoff patterns [2,3]. Decreased precipitation in certain regions of Africa, Asia and in the Mediterranean, especially during the summer months, together with greater evapotranspiration loss with higher temperatures and an increasing anthropogenic water demand, are expected to reduce stream discharge [4]. Therefore, flow intermittency might increase in both Mediterranean and more temperate regions [5,6].

Flow cessation reduces habitat availability and alters habitat characteristics [7]. Oxygen levels in water decrease as temperature, sedimentation and nutrient concentrations rise [8]. Although increased temperature is expected to accelerate microbial colonization and activity [9,10], the simultaneous increase in leaching and decline in water quality might also constrain microbial density and activity [11,12], thereby promoting antagonistic impacts on the quality of the basal resources. Aquatic hyphomycetes are more nutrient-enriched than the leaf tissue they colonize [13,14] and by means of their enzymatic activities, they can immobilize dissolve nutrients from water and transform recalcitrant polymers into more labile molecules [13,15,16]. In addition, shifts in water nutrient ratios will also likely affect microbial activity and production [17,18], potentially altering resource stoichiometry [19]. Therefore, determining the effects of drought on microbial compartments and basal resources is necessary to understand and scale-up the bottom-up effects of these changes on consumers.

Increases in temperature will also directly affect ectothermic consumers by increasing their metabolic rates and altering many of their biological processes, such as development time, respiration, growth and consumption [20,21]. However, the rate of change might be different among processes and further constrained by resource quality [22]. Therefore, if the effects of drought on consumer resources do not ensure a balanced supply of key elements, there may be a mismatch between consumers’ metabolic demands and the availability of the resources to meet them. Consumers use different behavioral or physiological processes to achieve the appropriate balance of elements required for maintenance, growth and reproduction [23,24]. For instance, nutrient limitations might be addressed by increasing the feeding rate or selecting more nutritious patches of food [24,25]. According to stoichiometric regulation, digestion and absorption may also be adjusted to favor the retention of the most limiting element, and excess nutrients can be released by excretion or respiration [26]. Consequently, the extent to which individuals will be able to compensate for this imbalance will ultimately determine the constraints on an individual’s performance and survival as well as alterations in the consumers’ nutrient cycling [23,25].

Most watercourses in temperate regions consist of small forested headwater streams, mainly fuelled by allochthonous organic matter [27], and thus a major fraction of energy in the ecosystem flows through the detritivore chain. Because large stoichiometric imbalances exist between shredders and the particulate organic matter they feed on [28], further changes in resource quality during flow intermittence [29] might have consequences for both consumers’ fitness and organic matter processing in streams. In this study, we specifically aim to evaluate (i) how resource quality will affect consumers’ growth, feeding, resource utilization and nutrient cycling and (ii) whether a temperature increase will attenuate or, alternatively, accentuate these changes. To this end, a laboratory experiment was performed by feeding the shredder *Echinogammarus berilloni* Catta with *Populus nigra* L. leaves that were previously exposed either to an intermittent or a permanent stream reach and incubating each of the two diet groups at two controlled temperatures (15 and 20°C). We hypothesized that the lower quality of leaves conditioned at the intermittent reach (*i*.*e*., resulting from lower microbial density and activity in pool-conditioned leaves) would result in compensatory feeding by the shredders and in alterations in their nutrient ratios in feces and excreta to favor retention of the most limiting nutrient. In contrast, higher temperatures should increase consumers’ metabolism, leading to higher consumption, growth, egestion and excretion rates. Consumers in higher temperatures that were also fed lower quality food must strike a balance between their increased energetic demands and the reduced resource quality. This can be accomplished by increasing their consumption rates and/or their assimilation efficiencies in favor of the most limiting nutrient, the later resulting in a higher release of the non-limiting nutrient. Finally, the extent to which organisms will be able to compensate, either by increasing consumption or modifying assimilation efficiencies, will determine the effects of changes in temperature and resource quality on the elemental composition of consumers.

## Materials and Methods

### Ethics statement

This study was performed in a natural park area managed by the Diputació de Barcelona, and the permit for the work was obtained from the staff of the park. This study did not involve endangered or protected species.

### Experimental setup

In a laboratory experiment, *Echinogammarus berilloni* Catta were fed leaf litter conditioned in an intermittent stream (INT) or leaf litter conditioned in a permanent stream (PERM). Individuals fed either type of leaves were incubated at either 15 or 20°C, resulting in 4 treatments: PERM15, INT15, PERM20 and INT20. Fifteen replicates (glass microcosms) were performed for each treatment. The experiment lasted 14 days in temperature-controlled incubators (F1, IBERCEX, Madrid, Spain).

Mature *E*. *berilloni* males were collected from Riera de Vallforners, a 2^nd^-order stream located within Montseny Natural Park (41° 42’ 25” N, 2° 21’ 06” E), by separating precopula pairs. Individuals were placed in 250 mL plastic containers, covered with 1-mm mesh net and introduced to a portable refrigerator filled with stream water for transport to the laboratory. In the laboratory, individuals were randomly split into two groups and acclimatized at 15 and 20°C under a 12 h light: 12 h dark photoperiod for one week and fed conditioned poplar litter *ad libitum*. Twenty-four hours prior the start of the experiment, the test animals were starved to allow evacuation of their gut contents. *E*. *berilloni* were photographed, and the dorsal length (DL) of their first thoracic segment measured (software: ImageJ v.1.45s, National Institutes of Health, USA; 0.001mm accuracy). *E*. *berilloni* body length (BL) was estimated using the equation BL = 14.458*DL—0.11 (*r* ^*2*^ = 0.969; *p*<0.001; *n* = 92; [30]).

For the experiment, *E*. *berilloni* were individually allocated to glass microcosms (8.5 cm diameter x 9 cm high) containing 250 mL of filtered stream water. Water was oxygenated for the duration of the experiment and replaced every 2 days to avoid ammonia accumulation and to compensate for water losses. Thirty individuals, previously acclimatized to the test temperatures (15 or 20°C), were randomly divided into the two food quality treatments (15 individuals per treatment).

### Leaf conditioning

*Populus nigra* leaves were conditioned at two selected reaches within La Tordera river catchment (NE Iberian Peninsula), a permanent stream reach (Santa Fe) and an intermittent stream reach (Fuirosos). The Tordera catchment is in a Mediterranean climate; most precipitation occurs in autumn and spring, summers are warm and dry, and winters are mild. However, the altitudinal difference between the study reaches led to different microclimates with contrasting temperature, precipitation and evapotranspiration (S1 Table), resulting in different hydrological regimes (for further details, see Von Schiller et al. [17]). The permanent stream (Santa Fe: 41° 46’ 48” N, 2° 27’ 15” E; 1189 m a.s.l.) is located in Montseny Natural Park. The studied riffle-pool reach is mostly flanked by beech (*Fagus sylvatica* L.), and stream flow is continuous throughout the year. The intermittent stream (Fuirosos: 41° 42’ 20” N, 2° 35’ 56” E; 106 m a.s.l.) is located in Montnegre-Corredor Natural Park, which has a warmer and drier climate. With the exception of the wettest years, stream discharge is intermittent during summer, when the streambed is dry for 2–3 months. The study reach is surrounded by dense riparian vegetation dominated by alder (*Alnus glutinosa* L.), hazel (*Corylus avellana* L.) and holm oak (*Quercus ilex* L.). Both reaches flow through a granitic substrate mainly composed of cobbles and sand.

Air-dried, stalkless *Populus nigra* L. leaves, collected in the Tordera catchment (41° 45’ 41” N, 2° 35’ 06” E) in autumn 2011, were assembled into groups of ~5 g and enclosed in 0.5-mm-mesh bags (25 x 30 cm). Leaf colonization started in June 2012 in both stream reaches, when the intermittent reach was still connected but had low flow. After 2 weeks (on June 26^th^), the flow was interrupted in the intermittent stream, and leaf colonization continued in an isolated pool for 3 more weeks. Differences in the physicochemical characteristics of the stream water between the stream reaches are summarized in S1 Table. Leaves were recovered on two consecutive weeks (10 leaf bags per reach and date) to provide fresh food to the study detritivores throughout the 2-week experiment. The leaves were transported in a cooler to the laboratory, where they were gently rinsed with distilled water. On each sampling date, 5 subsamples were randomly taken from 5 different leaf bags for the analysis of chemical quality and microbial biomass. Samples for ergosterol determination (5 12-mm diameter poplar discs) were placed in plastic vials and frozen (-20°C) until analysis, and samples for bacterial density analyses (1 12-mm diameter poplar disc) were stored in sterilized glass vials with filtered stream water (0.2 μm nitrocellulose, Whatman) fixed with formalin (2%). Biofilm from a known leaf surface was scraped with a toothbrush, filtered on glass fiber filters (Whatman GF/F) and frozen at—20°C until chlorophyll *a* analysis. An extra set of 5 12-mm diameter poplar discs and the remaining poplar leaves were lyophilized (excluding the main vein) and weighed to the nearest 0.01 mg. The leaf discs were ashed for 4 h at 450°C and reweighed to obtain the ash-free dry mass (AFDM). Lyophilized leaves were ground and used for chemical analyses.

### Invertebrate consumption, growth, egestion and excretion

Fifteen premeasured *E*. *berilloni* per treatment were fed *ad libitum* with 20 12-mm diameter preconditioned (PERM or INT) poplar discs at either 15 or 20°C. Four extra microcosms per treatment, containing 20 leaf discs but no animals, were used as a control for leaf mass losses other than consumption. After one week, the leaf material was removed and replaced by 20 fresh conditioned leaf circles. The removed leaf discs were collected, dried at 60°C until constant weight and weighed to the nearest 0.01 mg. The leaf dry mass (DM) that was offered to the invertebrates was estimated from extra sets of conditioned (PERM and INT) leaves. At the end of the experiment, individuals were measured again, freeze-dried, weighed to the nearest 0.001 mg, ground and individually used for the carbon (C), nitrogen (N), phosphorus (P), protein and lipid content evaluation as described below.

Consumption was calculated as the loss of leaf DM corrected by the DM loss in the control microcosms of the respective treatments. The relative consumption rate (*RCR*) was calculated as *RCR = Consumption/(DM*_*cons*_ * *day*), where *DM*_*cons*_ is the *consumers*’ dry mass (g) at the end of the experiment and *day* is the number of days the test lasted. The rates of C, N and P consumption were determined by multiplying the C, N and P content of the offered resources. Individuals’ relative growth rates (*RGR*) were estimated as *RGR = (BL*_*f*-_ *BL*_*i*_)/(BL * day), where *BL*_*f*_ and *BL*_*i*_ are the final and initial body length (mm), respectively; *BL* is the mean *body length* between the start and the end of the test; and *day* is the number of days the test lasted. Survivorship was also registered every other day during all the experiment.

Feces produced during the feeding experiment were removed every 3–4 days with a Pasteur pipette and pooled for each individual on a pre-weighed vial, freeze dried and weighed to the nearest 0.01 mg. A subsample was used to determine C, N and P %. Relative egestion rates were calculated in terms of the egested DM divided by the individual’s dry mass per day. The rates of C, N and P egestion were determined by multiplying the C, N and P % of each individual egesta.

Excretion rates were calculated on the 11^th^ day after the start of the experiment. Individuals were rinsed with UV-purified water and introduced into sterile Falcon tubes containing 50 mL of UV-purified water. The incubation took place in darkness, at either 15 or 20°C according to the treatment, over 4 h. Water samples were collected to determine the total dissolved phosphorus, nitrogen and organic carbon (TDP, TDN, DOC, respectively) at the start and end of the incubation. All water samples were filtered with pre-combusted glass fiber filters (Whatman GF/F) and immediately frozen (–20°C) until analysis. Falcon tubes containing UV-purified water but no animals served as blanks. Relative excretion rates were calculated in terms of mg of the element excreted (corrected by blanks) divided by individual’s dry mass per h.

### Microbial biomass

Fungal biomass in conditioned *Populus* leaves was measured from ergosterol analysis. Ergosterol was extracted from leaf disks using KOH methanol 0.14 M at 80°C for 30 min and then separated by solid-phase extraction (Waters *Sep-Pack* Vac RC, 500 mg, tC18 cartridges, Waters Corp., Milford, MA, USA; [31]). Ergosterol was quantified using an HPLC-MS/MS (HPLC Agilent 1100 series, Waldbronn, Germany) [32] equipped with an API 3000 triple-quadrupole mass spectrometer (PE Sciex, Concord, ON, Canada). The mobile phase was 100% methanol at a flow rate of 450 μL min^-1^. Separation was achieved with a Luna 5 μm c18, 100A, 150 x 2 mm analytical column (Phenomenex, Torrance, CA, USA). Quantification was performed with a multiple reaction monitoring (MRM) method at *m/z* 379.1/69.1, and the conversion to C % of fungal biomass was achieved using a conversion factor of 5.5 μg ergosterol mg^-1^ fungal dry mass [33] and 43% C content in fungal dry mass [34].

To estimate bacterial density, fixed samples were sonicated (1 + 1 min, Selecta, 40 W power, 40 kHz frequency) and agitated for 30 min after dilution with pyrophosphate (1:10). Subsequently, the samples were stained for 20 min with 4,6-diamidino-2- phenylindole (DAPI; final concentration of 2 μg mL^-1^) and filtered through 0.2 μm irgalan black-stained polycarbonate filters (Nuclepore, Whatman). Bacteria were then counted using a fluorescence microscope (Nikon, Tokyo, Japan) under 1250x magnification. Fifty random fields per filter were counted. The bacterial biomass in terms of carbon was estimated based on a conversion factor of 2.2 · 10^-13^ g C·μm^-3^ [35] and considering a mean bacterial biovolume of 0.1 μm^3^ [36].

Chlorophyll *a* was extracted in 90% acetone for 12 h in the dark at 4°C after 2 min of sonication (Selecta sonication bath at 150 W and 50 Hz). Then, the samples were further sonicated to ensure complete chlorophyll extraction. After filtration (GF/C, Whatman) of the extract, the chlorophyll concentration was determined spectrophotometrically (Lambda 2 UV/VIS spectrophotometer, Perkin-Elmer) following [37]. Algal biomass in terms of carbon was calculated assuming a carbon:chlorophyll-*a* ratio of 60 [38].

### Chemical analyses

The DOC and TDN were measured using a high catalytic oxidation Shimadzu TOC 5000 analyzer (Kyoto, Japan) with a coupled TN analyzer unit. The total C and N contents in the leaves, animals and feces were analyzed with a Thermo Elemental Analyzer 1108 (Thermo Scientific, Milan, Italy). The total P concentration was determined after a basic digestion (NaOH) of samples in an autoclave (110°C for 90 min) [39], and the posterior determination of the total phosphate concentrations (SRP) was performed using the ascorbic acid method [40]. The total phenolics were quantified by means of a Folin-Ciocalteau assay [41]. For the lipid content analyses, samples were homogenized using an ultrasonic homogenizer (200 W, 24 kHz; Hielscher UltrasonicsGmbH, Teltow, Germany), and the lipids were extracted with a mixture of chloroform and methanol (2:1) according to Bligh and Dyer [42]. The total lipid content was analyzed using the colorimetric sulphophosphovanillin method [43]. Protein extraction followed Baerlocher et al. [44], and the quantification was performed using the Bradford assay. The extraction and determination of soluble carbohydrates was performed according to Mansfield and Bärlocher [45].

### Data analysis

The assimilation efficiencies (AE; for C, N and P) were calculated as the percentage ratio between digested (ingested-egested) and ingested food. The N and P internal mass balances were estimated as the arithmetic difference between ingested and egested+excreted elements after correcting the values for time and individual dry mass according to Díaz Villanueva et al. [46].

Leaf microbial biomass and quality was compared between conditioning treatments (PERM versus INT) and weeks with a two-way analysis of variance (2-way ANOVA). When neither *week* nor the interaction term were significant, a *t*-test considering only the conditioning treatment was used instead.

The effects of leaf quality and temperature (fixed effects) on the response variables (i.e., RCRs, RGRs, assimilation efficiencies, egestion and excretion rates, egested and excreted ratios, internal mass balances and consumers’ stoichiometry and lipid and protein concentration) were analyzed by an analysis of covariance (2-way ANCOVA) using consumers’ dry mass (DM) as the covariate. We started with the most complex model, introducing all of the possible interactions (including interactions of covariates x factors, following García-Berthou and Moreno-Amich [47]). The general linear model was simplified by removing non-significant interactions (*P*>0.100). When the covariate was not significant, it was also removed from the model, and an ANOVA was used instead. The ANCOVA was also used to compare the condition of the consumers (body mass relative to body length) at the end of the experiment using the invertebrate DM as a fixed factor and its BL as the covariate [48]. To test whether the N and P mass balances significantly deviated from 0, a one-sample Student’s *t*-test was used.

When significant differences were found, pairwise comparisons adjusted using the Dunn-Sidak correction were performed to determine the differences among the 4 treatment means [49]. Data were log or arcsine transformed when necessary to meet the assumptions of normality and equal variance. All statistical analyses were performed with IBM SPSS statistics 20.0 for Windows (SPSS Inc., Chicago, IL, USA).

## Results

### Conditioned leaf quality

Fungal C content was higher in PERM leaves, whereas bacterial C content was higher in INT leaves (Table 1). There was no difference in algal C content between INT and PERM leaves (Table 1). Leaves conditioned in the PERM reach had higher soluble carbohydrates, protein and phenol concentrations (Table 1), lower C:P and N:P ratios and higher P concentrations, especially in the second week (Table 1, *Conditioning* x *Week* interaction). The % C was higher in leaves conditioned in the INT reach, whereas there were no differences in the C:N ratios or % N among the treatments (Table 1, *Conditioning effect*). The % C and N increased on the second week, leading to higher C:P and N:P ratios but lower C:N ratios (Table 1, *Week effect*). There were no differences in the leaves’ lipid concentrations among the treatments (Table 1).

**Table 1:** Microbial biomass, chemical composition (mean ± SE) and summary statistics of poplar leaves conditioned in an intermittent (INT) and permanent (PERM) reach.

		PERM reach	INT reach	*Conditioning*		*Week*		*Conditioning* x *Week*	
		*Mean (± SE)*	*Mean (± SE)*	*t*_*18*_ / *F*_(*1*, *16*)_	*P*	*F*_(*1*, *16*)_	*P*	*F*_(*1*, *16*)_	*P*
*Microbial composition*	Bacterial C (mg g^-1^AFDM)	0.141 (± 0.013)	0.369 (± 0.078)	-2.885	**0.010**	-	-	-	-
	Fungal C (mg g^-1^ AFDM)	31.730 (± 2.130)	13.780 (± 0.870)	7.789	**<0.001**	-	-	-	-
	Algal C (mg g^-1^ AFDM)	0.91 (± 0.26)	1.36 (± 0.38)	-1.319	0.205	-	-	-	-
*Chemical composition*	Lipids (mg g^-1^ AFDM)	40.00 (± 3.85)	33.92 (± 3.08)	1.215	0.241	-	-	-	-
	Proteins (mg g^-1^ AFDM)	74.76 (± 4.83)	52.49 (± 4.07)	3.523	**0.002**	-	-	-	-
	Soluble carbohydrates (mg g^-1^ AFDM)	2.26 (± 0.23)	1.61 (± 0.17)	2.312	**0.033**	-	-	-	-
	Phenols (mg g^-1^ AFDM)	13.43 (± 0.70)	7.98 (± 0.35)	6.943	**<0.001**	-	-	-	-
	C (%)	51.40 (± 0.41)	52.45 (± 0.44)	8.923	**0.009**	6.821	**0.020**	0.074	0.790
	N (%)	1.48 (± 0.05)	1.42 (± 0.03)	2.539	0.131	20.124	**0.000**	1.189	0.292
	P (%)	0.058 (± 0.001)	0.053 (± 0.003)	6.419	**0.022**	3.674	*0*.*073*	12.708	**0.003**
	C:N	40.95 (± 1.43)	43.34 (± 0.90)	2.685	0.121	7.165	**0.017**	0.923	0.351
	C:P	2291.40 (± 54.99)	2627.78 (± 140.61)	10.100	**0.006**	6.963	**0.018**	13.665	**0.002**
	N:P	56.23 (± 1.24)	60.94 (± 3.68)	4.703	**0.046**	29.911	**<0.001**	11.825	**0.003**

The results are the mean (± SE) for the different treatments.

Statistical results derived from a 2-way ANOVA or *t*-test after the non-significant effects of the week and interactions were removed (*P*>0.100).

*P* values <0.050 are indicated in bold, and *p* values <0.100 are shown in italics.

All ratios are molar ratios.

### Consumption, egestion and assimilation efficiencies

**Fig 1:**
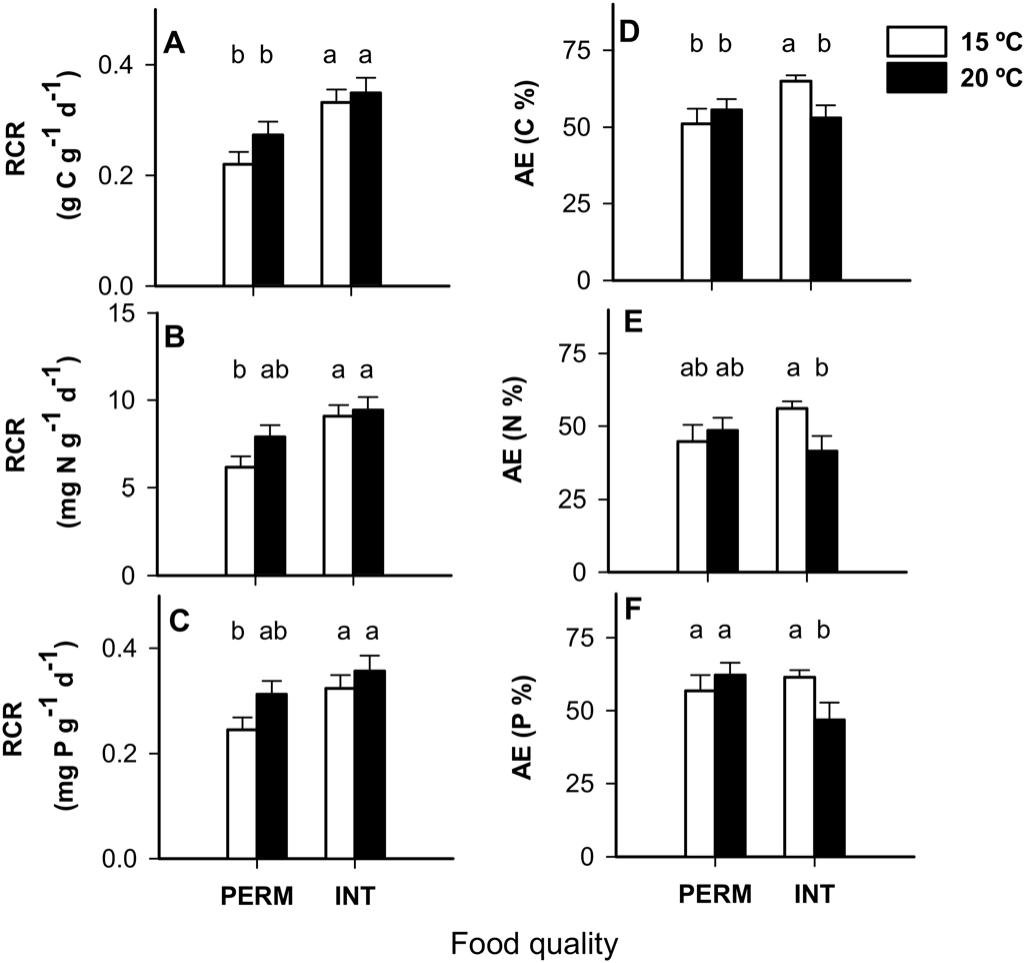
Relative consumption rate (RCR; A-C) and assimilation efficiency (AE; D-F) for *E*. *berilloni* (n = 11–15) maintained at 15 and 20°C and fed PERM and INT leaves. The results are the mean (± SE) for the different treatments. The least-square means are shown for *RCR* to correct for the DM effect (ANCOVAs, *P*<0.100). Different letters indicate significant differences based on independent pairwise comparisons adjusted by the Dunn-Sidak correction at α = 0.05.

*E*. *berilloni* fed INT leaves had higher C, N and P RCRs (Table 2, *Leaf quality effect*; Fig. 1A-C) but also higher N and P egestion rates (Table 2 and S2 Table, *Leaf quality effect*). Egestion rates were higher at 20°C for all of the elements analyzed (Table 2 and S2 Table, *Temp effect*). Consequently, the AE significantly decreased with temperature when the individuals were fed INT leaves, whereas no effect of temperature occurred when they were fed PERM leaves (Table 2, *Leaf quality x Temp effect*; Fig. 1D-F). The individuals’ consumption and egestion rates diminished with the individuals’ DM (Table 2, *DM effect*).

**Table 2:** ANCOVA or ANOVA results for the effects of leaf quality and temperature on *E*. *berilloni* consumption, egestion and excretion rates and ratios, digestion efficiency and net gain.

	Consumption rate		Egestion rate		Digestion efficiency		Excretion rate		Net gain			Feces ratio		Excreta ratio	
	*F*_(*1*, *48*)_	*P*	*F*_(*1*, *48*)_	*P*	*F*_(*1*, *47*)_	*P*	*F*_(*1*, *47*)_ ^*1*^	*P*	*F*_(*1*, *44*)_	*P*		*F*_(*1*, *47*)_	*P*	_(*1*, *39*)_ ^*2*^	*P*
**C**											**C:N**				
*Leaf quality*	15.195	**<0.001**	3.237	*0.078*	2.132	0.151	0.103	0.750	-	-		16.539	**<0.001**	0.040	0.842
*Temp*	2.050	0.159	5.297	**0.026**	0.871	0.355	0.013	0.911	-	-		1.285	0.263	0.467	0.498
Leaf quality*Temp	0.547	0.463	0.992	0.324	4.832	**0.033**	1.140	0.292	-	-		9.713	**0.003**	0.007	0.932
*DM (covar*)	25.106	**<0.001**	14.246	**<0.001**	-	-	-	-	-	-		8.911	**0.004**	-	-
**N**											**C:P**				
*Leaf quality*	11.228	**0.002**	5.657	**0.021**	0.230	0.634	0.000	0.991	1.380	0.247		8.991	**0.004**	1.594	0.214
*Temp*	2.391	0.129	6.594	**0.013**	1.212	0.277	2.035	0.160	1.236	0.272		0.366	0.548	1.089	0.303
Leaf quality*Temp	1.073	0.306	0.452	0.505	4.299	**0.044**	5.524	**0.023**	10.590	**0.002**		3.875	*0.055*	7.619	**0.009**
*DM (covar*)	25.167	**<0.001**	17.416	**<0.001**	-	-	-	-	-	-		25.176	**<0.001**	-	-
**P**											**N:P**				
*Leaf quality*	5.721	**0.021**	9.803	**0.003**	1.527	0.223	7.442	**0.009**	3.894	*0.055*		4.101	**0.049**	3.977	*0.052*
*Temp*	3.650	*0.062*	4.720	**0.035**	0.855	0.360	1.037	0.314	1.892	0.176		0.745	0.392	0.138	0.712
Leaf quality*Temp	0.432	0.514	3.484	*0.068*	5.030	**0.030**	2.724	0.106	0.126	0.724		8.559	**0.005**	6.707	**0.013**
*DM* (*covar*)	24.477	**<0.001**	25.861	**<0.001**	-	-	-	-	-	-		20.081	**<0.001**	-	-

ANOVAs were used after the non-significant (*P*>0.100) covariable (*DM*) was excluded from the model.

*P* values <0.050 are indicated in bold, and *p* values <0.100 are shown in italics. Temp stands for temperature and *Covar* stands for covariable.

C net gain was not analyzed because respiration had not been measured.

^1^ (1, 40) for C excretion rates.

^2^ (1, 39) for C:N and C:P ratios in excreta.

**Fig 2:**
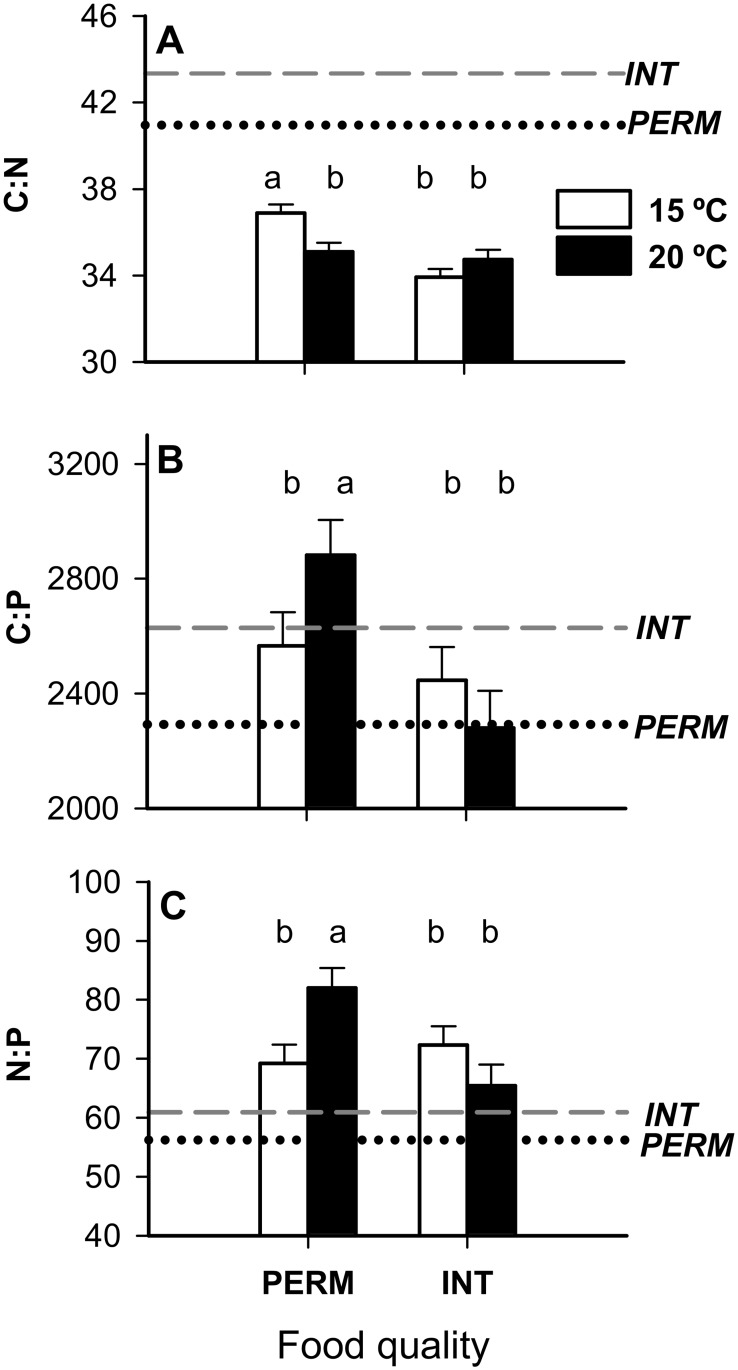
Molar ratios in feces of *E*. *berilloni* (n = 11–15) maintained at 15 and 20°C and fed PERM and INT leaves. The results are the mean (± SE) for the different treatments. The least-square means are shown to correct for the DM effect (ANCOVAs, *P*<0.100). The horizontal lines represent molar ratios of leaves conditioned under PERM (gray dashed line) and INT (black dotted line) conditions. Different letters indicate significant differences based on independent pairwise comparisons adjusted by the Dunn-Sidak correction at α = 0.05.

All of the molar ratios in the feces depended on the interaction between food quality and temperature: the C:N ratios were significantly higher when individuals had been reared at 15°C and fed PERM leaves, whereas the C:P and N:P ratios were higher at 20°C under the same diet (Table 2, *Leaf quality x Temp effect*; Fig. 2A-C). The C:N ratios in feces were lower than in the bulk leaf material, indicating that C was more efficiently assimilated than N in all of the treatments (Fig. 2A). Interestingly, this response differed between treatments for P. There were higher C:P and N:P ratios in the feces than leaves when the individuals were fed PERM leaves, which suggests preferential assimilation of P over C and N in the PERM treatments. However, under an INT diet, the individuals preferentially assimilated C over P, especially at 20°C, and they only assimilated P more efficiently than N at 15°C (Fig. 2B, C). The ratios in the feces increased with the individuals’ DM (Table 2, *DM effect*).

### Excretion rates and elemental mass balances

**Fig 3:**
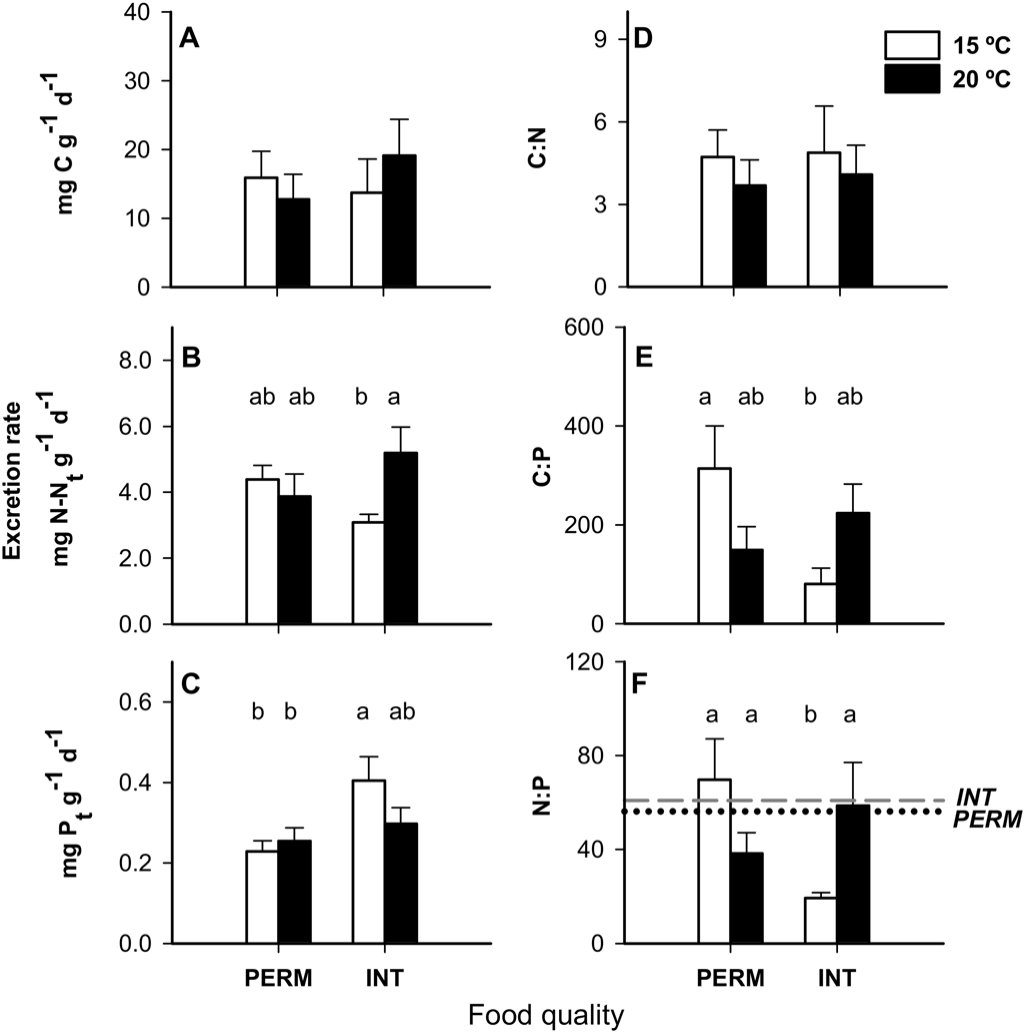
Relative excretion rate (A-C) and molar ratios in excreta (D-F) of *E*. *berilloni* (n = 11–15) maintained at 15 and 20°C and fed PERM and INT leaves. The results are the mean (± SE) for the different treatments. The horizontal lines represent molar ratios of leaves conditioned under PERM (gray dashed line) and INT (black dotted line) conditions. The C:N and C:P ratios of leaves fell above the range of the graphs. Different letters indicate significant differences based on independent pairwise comparisons adjusted by the Dunn-Sidak correction at α = 0.05.

The N excretion rates increased with temperature when *E*. *berilloni* were fed INT leaves (Table 2, *Leaf quality x Temp effect*; Fig. 3B), whereas the P excretion rates were higher for the INT treatments irrespective of temperature (Table 2, *Leaf quality effect*; Fig. 3C). The C:P and N:P ratios in excreta were significantly lower with an INT diet at 15°C (Table 2, *Leaf quality x Temp effect*; Fig. 3E-F), which also resulted in lower N:P ratios in the excreta than in the leaves. There were no differences in C excretion rates or C:N ratios in the excreta between treatments (Table 2; Fig. 3A-D).

**Fig 4:**
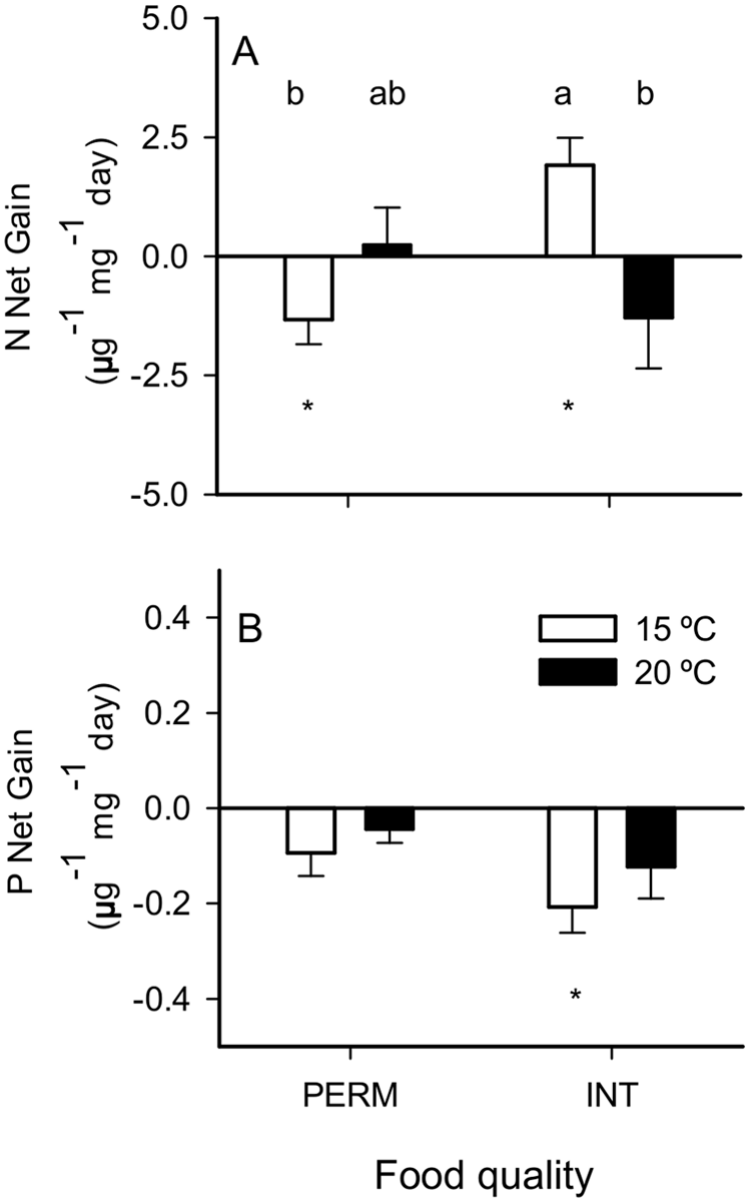
Net gain of (A) N and (B) P by *E*. *berilloni* (n = 11–15) maintained at 15 and 20°C and fed PERM and INT leaves. The results are the mean (± SE) for the different treatments. Different letters indicate significant differences based on independent pairwise comparisons adjusted by the Dunn-Sidak correction at α = 0.05. * Net gain was significantly different from 0 (*t*-test, *P*<0.050).

When the individuals were fed INT leaves, the N mass balance decreased significantly at warmer temperatures, shifting from a positive balance at 15°C to a null balance at 20°C (Table 2, *Leaf quality x Temp effect*; Fig. 4A). However, when individuals were fed PERM leaves, they shifted from a negative balance at 15°C to a null balance at 20°C, but the differences between temperatures were not significant in the PERM diets (Table 2, *Leaf quality x Temp effect*; Fig. 4A). P mass balances were not significantly different among the treatments (Table 2), although they tended to be lower in the INT treatments (Table 2, *Leaf quality effect*; Fig. 4B). The P mass balance was null for all of the treatments except for INT15, whose individuals loss P.

### Consumer stoichiometry and chemical content

*E*. *berilloni* had a lower N concentration when maintained at 20°C versus 15°C and when fed INT leaves versus PERM leaves (Tables 3 and 4, *Leaf quality* and *Temp effect*). In contrast, under an INT diet, individuals had lower P concentrations at 15°C (Tables 3 and 4, *Leaf quality x Temp effect*). Accordingly, the lowest C:P and N:P ratios and highest C:N ratios were found in individuals fed INT leaves at 20°C (Table 4). In addition, the P concentration of *E*. *berilloni* increased with individual biomass when fed PERM leaves (*P* = 0.022) but did not change under an INT diet (*P*>0.050; Table 4, *Leaf quality x DM effect*; S1 Fig.).

**Table 3:** Chemical composition (mean ± SE) of *E*. *berilloni* (n = 11–15) at the end of the experiment (14 days).

*Temp*	*Food quality*	C (%)	N (%)	P (%)	C:N	C:P	N:P	Lipids (mg g^-1^ DM)	Proteins (mg g^-1^ DM)
**15°C**	**PERM**	40.93 (±0.97)	7.59 (±0.02)^a^	0.56 (±0.04)^ab^	6.34 (±0.14)^b^	207.27 (±14.84)^ab^	33.29 (±1.66)^a^	51.45 (±5.78)^b^	293.70 (±18.00)
	**INT**	40.16 (±1.28)	7.07 (±0.22)^ab^	0.48 (±0.04)^b^	6.76 (±0.32)^b^	227.56 (±14.84)^a^	33.41 (±1.58)^a^	51.04 (±5.93)^b^	223.00 (±21.87)
**20°C**	**PERM**	39.03 (±0.78)	6.85 (±0.23)^b^	0.62 (±0.04)^ab^	6.69 (±0.19)^b^	178.64 (±15.35)^ab^	25.41 (±1.77)^b^	84.57 (±15.44)^b^	235.57 (±18.62)
	**INT**	42.52 (±1.56)	6.46 (±0.25)^b^	0.67 (±0.05)^a^	7.80 (±0.44)^a^	173.78 (±18.52)^b^	22.17 (±1.99)^b^	167.71 (±23.71)^a^	230.18 (±17.59)

The results are the mean (± SE) for the different treatments.

Least-square means are shown for the P (%), C:P and N:P values in *E*. *berilloni* to correct for the DM effect (ANCOVAs, *P*<0.100).

Within each column, treatments followed by ^a^ indicate significantly higher mean values than in treatments followed by ^b^, whereas ^ab^ denotes mean values are not significantly different from values followed by ^a^ or ^b^.

All ratios are molar ratios.

**Table 4:** ANCOVA or ANOVA results for effects of leaf quality and temperature on *E*. *berilloni* (n = 11–15) chemical composition at the end of the experiment (14 days).

	C (%)		N (%)		P (%)		C:N		C:P		N:P		Lipids (mg g^-1^ DM)		Proteins (mg g^-1^ DM)	
*Factors*	*F*_(*1*, *49*)_	*P*	*F*_(*1*, *47*)_	*P*	*F*_(*1*, *44*)_	*P*	*F*_(*1*, *47*)_	*P*	*F*_(*1*, *44*)_	*P*	*F*_(*1*, *42*)_	*P*	*F*_(*1*, *49*)_	*P*	*F*_(*1*, *47*)_	*P*
*Leaf quality*	1.383	0.245	3.833	*0.056*	5.483	**0.024**	6.267	**0.016**	2.775	0.103	7.889	**0.008**	7.206	**0.010**	3.922	*0.054*
*Temp*	0.038	0.705	8.239	**0.006**	7.973	**0.007**	6.013	**0.018**	10.248	**0.003**	14.279	**<0.001**	30.362	**<0.001**	1.760	0.191
Leaf quality*Temp	3.383	*0.072*	0.077	0.783	3.998	*0.052*	1.355	0.250	2.056	0.159	0.863	0.358	8.317	**0.006**	2.891	*0.096*
*DM (covar)*	-	-	-	-	2.118	0.153	-	-	3.712	*0.061*	6.115	**0.018**	-	-	-	-
Leaf quality* DM (covar)	-	-	-	-	6.442	**0.015**	-	-	3.290	*0.077*	7.221	**0.010**	-	-	-	-
Temp* DM (covar)	-	-	-	-	3.000	*0.090*	-	-	5.388	**0.025**	4.304	**0.044**	-	-	-	-
Leaf quality*Temp* DM (covar)	-	-	-	-	2.585	0.115	-	-	1.439	0.237	0.422	0.520	-	-	-	-

ANOVAs were used after the non-significant (*P*>0.100) covariable (*DM*) was excluded from the model.

*P* values <0.050 are indicated in bold, and *p* values <0.100 are shown in italics. *Temp* stands for temperature and *Covar* stands for covariable.

The lipid concentration was significantly higher for the INT20 individuals (Tables 3 and 4, *Leaf quality x Temp effect*), whereas the protein concentration was not affected by food quality or temperature, despite a tendency to be lower in the INT versus PERM treatments, especially at 15°C (Tables 3 and 4, *Leaf quality* and *Leaf quality x Temp effects*).

### Growth, condition and survival

**Fig 5:**
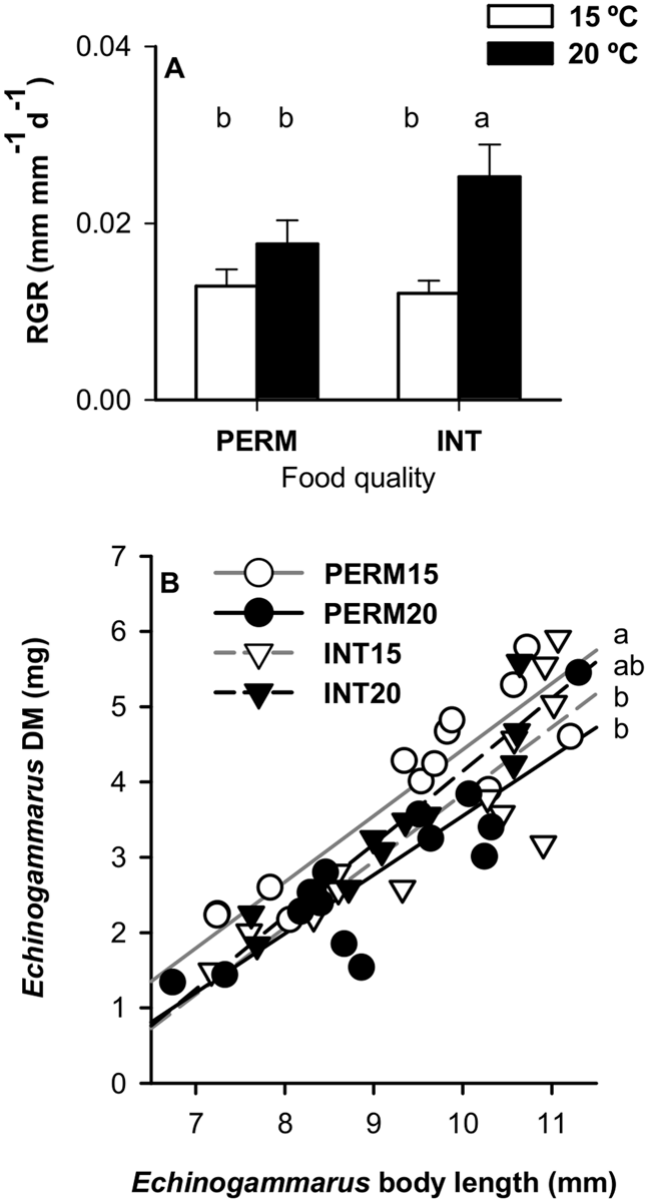
Relative growth rate (RGR, mean ± SE) and (B) condition of *E*. *berilloni* (n = 11–15) maintained at 15 and 20°C and fed PERM and INT leaves. **(A)** Linear regressions (*P*<0.001) are shown for each treatment; the *r* ^2^ values were 0.825, 0.751, 0.744 and 0.888 for PERM15, IN15, PERM20 and INT20, respectively. Different letters indicate significant differences based on independent pairwise comparisons adjusted by the Dunn-Sidak correction at α = 0.05.

The RGRs were higher for individuals maintained at 20°C when fed INT leaves (ANOVA, *Leaf quality x Temp effect*, *F*_1, 48_ = 2.825, *P* = 0.099; *Temp effect*, *F*_1, 48_ = 12.949, *P* = 0.001; *Leaf quality effect*, *F*_1, 48_ = 1.876, *P* = 0.117; Fig. 5A). The condition of individuals depended on the interaction between leaf quality and temperature (ANCOVA, *Leaf quality x Temp effect*, *F*_1, 47_ = 11.252, *P* = 0.002, *Size effect*, *F*_1, 47_ = 182.307, *P*<0.001; Fig. 5B), and condition was better for PERM15 than for INT15 or PERM 20 individuals (Fig. 5B, post-hoc multiple comparison test). Survival was 100% for PERM15, 93% for INT15 and PERM20 and 80% for INT20. Mortality started on the third day in the INT treatments but did not occur until day 12 in the PERM treatments.

## Discussion

This study shows that the assimilation of P is constrained when *E*. *berilloni* are fed lower quality leaves (*i*.*e*., leaves with higher C:P ratios). Higher consumption rates under such a lower quality diet compensated for lower P assimilation at 15°C but not at 20°C. However, despite its lower nutrient assimilation, growth rates were higher at warmer temperatures under a lower quality diet. This mismatch might be responsible for the observed changes in consumers’ body tissue composition and increased mortality under this treatment, which suggests more severe consequences for *E*. *berilloni* feeding on less nutritious resources under the expected climate warming scenario [1].

*E*. *berilloni* that were fed leaves conditioned in the intermittent stream (INT leaves) showed an increase in relative consumption rates but also higher nitrogen egestion and phosphorus egestion and excretion. The metabolic changes in the consumer might be linked to specific changes in leaf quality. In this study, the INT leaves were of lower quality, containing less protein, soluble carbohydrate and phosphorous than the PERM leaves. Resource conditioning in the intermittent stream pools increased the C:P and N:P ratios in leaves, and a diet of these leaves may be P limiting in higher trophic levels [16,23,50]. Previous studies in the same stream found high phosphatase activity in the biofilms of summer pools, which underscores the P limitation in the drying phase [12,19]. More intense leaching and deoxygenation in pools [7,51], especially among the leaf packs, might have constrained the fungal growth [52,53] and protein accrual [14], whereas higher temperatures and intense leaching might have reduced both the phenol and soluble carbohydrate concentrations of the leaves [11,54]. Similar changes in leaf quality caused by anoxic summer conditions were reported by Dieter et al. [53], who found a decrease in phenolics and P content but also an increase in lignin and cellulose content, which further reduced the quality of the leaves as a food resource for consumers. Therefore, the higher relative consumption rates when *E*. *berilloni* were fed INT leaves may reflect compensatory feeding resulting from the lower resource quality (*i*.*e*., lower P %, protein and soluble carbohydrate content). Such compensation in feeding rates has been widely described for many herbivores [23,55,56]. For detritivores, compensatory feeding has been observed for low quality food, with lower nutrient concentrations and higher content of recalcitrant compounds (*i*.*e*. lignin, tannins) when food quantity is not limiting [16,57,58]. In the natural stream ecosystem, the availability of detritus in the streambed is influenced by the timing of inputs from the riparian forest as well as the hydrology [59–61]. When the flow ceases, the transport of organic matter downstream also declines, and it accumulates in pools [62,63]. Furthermore, hydric stress causes earlier leaf abscission to riparian vegetation, and the greatest leaf fall coincides with the cessation of flow [60]. Therefore, despite the lower nutritional quality of pool-conditioned leaves, the high abundance of this resource might enable compensatory feeding in shredders in intermittent systems.

A diet of INT leaves also caused a greater loss of nutrients through egestion. Differences in the stoichiometry between feces and consumed leaves indicate *E*. *berilloni* assimilated P more efficiently than N and C when fed PERM leaves. However, the capacity to preferentially assimilate P over other elements was lost under an INT diet, suggesting that leaves conditioned in pools were not only more P-depleted, but that they also sequestered P in molecules that constrained their assimilation by *E*. *berilloni*. Changes in the fatty acid composition and essential amino acid abundance of leaves have been described during the drying phase in this intermittent stream [64,65]. Some studies indicate shredders can complement their diet with feces [66–69], especially if juveniles [66–68]. However, a recent study indicated that unlike in other sympatric freshwater gammaridean species, the morphology of the feeding structures in *E*. *berilloni* would not favor coprophagy [70]. Indeed, we did not observe feeding on fecal pellets during our experiment and furthermore, the compensatory feeding and nutrient-enriched ratios in feces under an INT diet suggest coprophagy has not been important in our experiment.

Increases in temperature affected the ability of *E*. *berilloni* to compensate for P assimilation efficiency. At 15°C, the loss of the absorptive regulatory capacity to preferentially assimilate P [23,24] was compensated for by the higher consumption rates, but this did not occur at 20°C. Egestion rates were also higher at warmer temperatures, which would have reduced the food gut passage time and contributed to the lower assimilation [26,71]. When fed PERM leaves, the individuals were able to compensate for these higher egestion rates at 20°C probably through higher digestive enzyme reactivity at warmer temperatures [20]. However, this did not occur under an INT diet, which resulted in lower assimilation efficiency in the INT20 treatment. Consequently, *E*. *berilloni* were not able to adjust their gut enzymatic activities to favor P assimilation when the temperature and food quality were simultaneously altered, potentially affecting their ability to meet their physiological requirements [72].

The inability of the INT20 individuals to preferentially assimilate P over N resulted in higher N excretion rates and N:P ratios in the excreta relative to the INT15 individuals, indicating post-absorptive stoichiometric regulation [24,26]. As a consequence, the consumers’ N net gain shifted from positive at INT15 to null at INT20, resulting in lower N concentrations in the *E*. *berilloni* that were reared at 20°C and fed INT leaves and a relative increase in % P. However, the N net balance was negative for PERM15, which is in contrast with the highest % N and protein concentration in the body tissue. Higher % N in leaves in the second week might have increased N losses through excreta, especially from PERM15 individuals, which were more efficient at assimilating N (*i*.*e*., higher C:N and lower N:P ratios in the feces, Fig. 2A). Interestingly, although *E*. *berilloni* fed INT leaves at 15°C could assimilate P more efficiently than N, they lost more P through excretion, which resulted in a net P loss and higher C:P ratios. A similar result was reported by Frost and Tuchman [73], who suggested a combination of high digestion but low absorption efficiencies, possibly due to increased concentrations of secondary compounds. This would have resulted in the digestion to dissolved forms of most of the ingested leaves but little incorporation of this material into new body mass [73]. Alternatively, if P had been assimilated in excess, *E*. *berilloni* would later release P through excreta to account for such an imbalance (post-absorptive regulation [24,26]). According to the growth rate hypothesis, which relates higher RGRs to higher amounts of P-rich rRNA [25,74], the lower RGR at 15°C than 20°C would explain the difference in P demands between temperatures under an INT diet. Consequently, the differences in consumer stoichiometry are not likely to be due to nutrient storage but rather to physiological responses to resource processing under conditions of lower food quality and higher temperatures (*i*.*e*., increased metabolism) [25]. This would be in agreement with Ferreira et al. [75] and Kendrick & Benstead [76], who also reported temperature-mediated effects on shredder caddisflies stoichiometry.

Higher temperatures and subsequent increases of metabolic rates increased the relative growth rate of *E*. *berilloni* but only for INT-fed individuals. This result agreed with Gracía & Pardo [77] who found higher detritivore growth rates when fed on lower quality leaves (eucalyptus *vs* alder) at warmer temperatures, but contrasts with experiments indicating more severe effects of lower food quality at warmer temperatures [76,78]. However, in most of these studies positively relating P content in food with shredder growth rates (*e*.*g*., [16,76,79,80]), increases in food quality were achieved through P additions, resulting in much larger differences between treatments than in our stream conditioning treatments. Furthermore, Kendrick & Benstead [76] also found detritivore growth rates increased at warmer temperatures in spite of lower diet quality, which they explained by a greater acquisition or assimilation of other growth-limiting factors than P at warmer temperatures. Although warrants further research, higher bacterial biomass in pool-conditioned leaves could have provided shredders with more essential polyunsaturated or bacterial fatty acids and contributed to the higher growth rates in the INT20 treatment [81].

The increase in *E*. *berilloni* body size at warmer temperatures under an INT diet did not translate into a better condition, probably because the higher metabolic demands at warmer temperatures were not compensated for by higher ingestion rates. This suggests that the higher lipid concentration in INT20 individuals did not correspond to an accumulation of storage lipids (*i*.*e*., triacylglycerols) as an energetic reserve that would otherwise have translated into higher % C. Therefore, the changes in body constituents appear to have responded to a greater conservation of lipids, especially structural lipids such as phospholipids, over other C-rich macromolecules, such as carbohydrates [26,82]. Amphipods preferentially store energy as glycogen, which enables the rapid mobilization of reserves [83]. Therefore, to satisfy their higher metabolic demands at warmer temperatures on a lower quality diet, *E*. *berilloni* would likely have metabolized their glycogen reserves while preserving structural lipids. This behavior could have caused the higher mortality observed in this treatment. Similarly, Danger et al. [16] and Flores et al. [58] related *shredder*’ mortality to differences in the biochemical quality of resources, which prevailed despite the occurrence of compensatory feeding for less nutritious food. Therefore, if this result is confirmed in longer term studies, the synergistic effects from the reduced food quality of leaves colonized in pools and higher metabolic demands at higher temperatures might result in a trade-off between growth and fitness that ultimately affects *Echinogammarus*’ survival.

In conclusion, our results showed that the effects of a lower quality diet on the physiology and stoichiometry of *E*. *berilloni* were accentuated at warmer temperatures, supporting previous results on a caddisfly physiology [46]. The reduced nutritional quality of the pool-conditioned leaves induced compensatory feeding in *E*. *berilloni*, but nutrient retention was lower under this scenario. Moreover, higher growth rates at warmer temperatures under a lower quality diet were not further compensated by increased consumption, assimilation efficiency or preferential assimilation of the most limiting nutrient. Instead, the demands shifted the shredders’ body stoichiometry and reduced their survival. Additional studies of the changes in macromolecular body composition might be helpful to explain changes in body stoichiometry [82] and to relate them to differences in fitness and survival. Finally, if these results are extended to other detritivores, the consequences of reduced leaf quality and increased temperatures for detritivore nutrient recycling might have broader ecosystem effects [84–86], resulting in more nutrient-rich resources for collector-gatherers and microbial leaf colonizers. However, the relevance of these effects to these trophic compartments might ultimately depend on how recalcitrant or digestible the egested and excreted compounds are for their target consumers.

## Supporting Information

S1 Fig Relationships between body dry mass and body P (%) of *E*. *berilloni* maintained at 15 and 20°C and fed PERM and INT leaves. Only significant (*P*<0.050) regressions are shown (PERM, log body P(%) = 0.015*DM* + 0.147, *r* ^2^ = 0.178, *P* = 0.022).
(TIF)

S1 Table Climatic and physicochemical characteristics of water in the study reaches. Mean (± SE) in the permanent (PERM) and intermittent (INT) reaches during the resource conditioning period (6^th^ June—26^th^ July 2012; n = 10).
(DOCX)

S2 Table Relative egestion rates of *E*. *berilloni*. C, N and P relative egestion rates (least-squares means ± SE) of *Echinogammarus* (n = 11–15) maintained at 15 and 20°C and fed PERM or INT leaves.
(DOCX)
