# Supplementary figures and images for: Consequences of Warming and Resource Quality on the Stoichiometry and Nutrient Cycling of a Stream Shredder
Source: PLoS One. 2015 Mar 4;10(3):e0118520. doi: 10.1371/journal.pone.0118520 (PMC4349742; doi:10.1371/journal.pone.0118520)

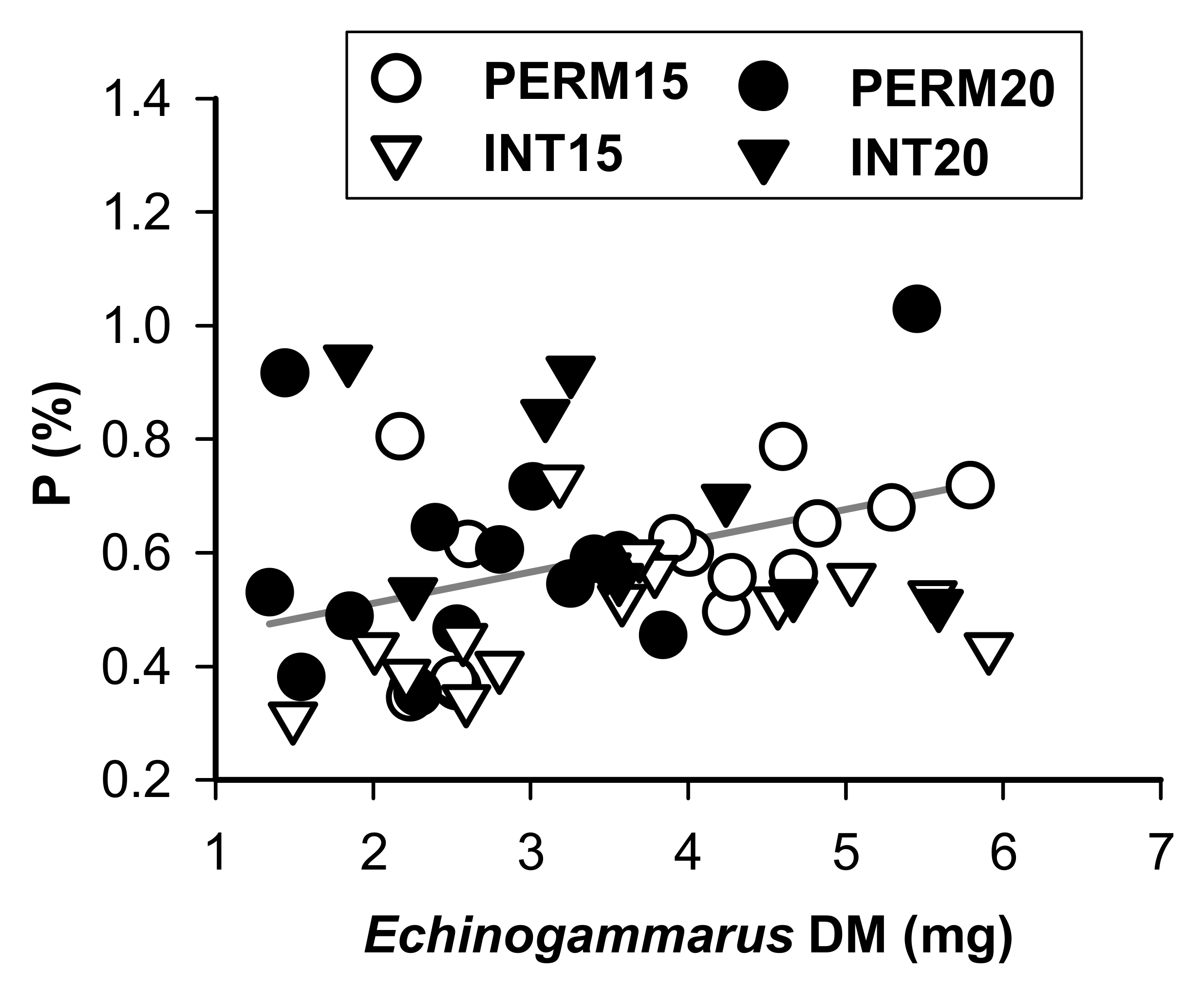

Supplement: S1 Fig — Only significant (P<0.050) regressions are shown (PERM, log body P(%) = 0.015DM + 0.147, r 2 = 0.178, P = 0.022). (TIF) [file pone.0118520.s001.tif]
