# Supplementary material for: Consequences of Warming and Resource Quality on the Stoichiometry and Nutrient Cycling of a Stream Shredder
Source: PLoS One. 2015 Mar 4;10(3):e0118520. doi: 10.1371/journal.pone.0118520 (PMC4349742; doi:10.1371/journal.pone.0118520)
Supplement: S1 Table — Mean (± SE) in the permanent (PERM) and intermittent (INT) reaches during the resource conditioning period (6th June—26th July 2012; n = 10). (DOCX) [file pone.0118520.s002.docx]

**Table S1.** **Climatic and** **physicochemical characteristics of water in the study reaches.** Mean (± SE) in the permanent (PERM) and intermittent (INT) reaches during the resource conditioning period (6^th^ June – 26^th^ July 2012; n=10).

| **Parameter** | **PERM reach** | | |  | **INT reach** | | | ***P value*** *(t-*test*)* |
| --- | --- | --- | --- | --- | --- | --- | --- | --- |
|  | Mean | ± | s.e. |  | Mean | ± | s.e. |  |
| Monthly mean air temperature (°C) (range) | 3 - 20^1^ |  |  |  | 4 – 28^2^ |  |  |  |
| Annual precipitation (mm) | 900^1^ |  |  |  | 650*^2^ |  |  |  |
| Conductivity (µS · cm^-1^) | 50.58 | ± | 0.92 |  | 317.11 | ± | 10.36 | **<0.001** |
| pH | 7.12 | ± | 0.03 |  | 7.22 | ± | 0.04 | 0.084 |
| O_2_ (mg/L) | 9.26 | ± | 0.08 |  | 3.61 | ± | 0.41 | **<0.001** |
| O_2_ (%) | 99.88 | ± | 0.49 |  | 39.88 | ± | 4.49 | **<0.001** |
| Temperature (°C) | 12.18 | ± | 0.30 |  | 19.14 | ± | 0.21 | **<0.001** |
| velocity (m ·s^-1^) | 0.026 | ± | 0.004 |  | 0.004 | ± | 0.001 | **<0.001** |
| Phenols (µg · L^-1^) | 0.271 | ± | 0.039 |  | 1.336 | ± | 0.171 | **<0.001** |
| DOC (mg · L^-1^) | 1.146 | ± | 0.046 |  | 4.592 | ± | 0.334 | **<0.001** |
| TN (mg · L^-1^) | 0.220 | ± | 0.012 |  | 0.571 | ± | 0.162 | **0.038** |
| Total inorganic N (mg · L^-1^) | 0.150 | ± | 0.011 |  | 0.249 | ± | 0.095 | 0.306 |
| N-NH_4_^+^ (mg · L^-1^) | 0.012 | ± | 0.002 |  | 0.217 | ± | 0.089 | **0.027** |
| N-NO_2_^-^ (mg · L^-1^) | 0.004 | ± | 0.000 |  | 0.004 | ± | 0.000 | 0.333 |
| N-NO_3_^-^ (mg · L^-1^) | 0.134 | ± | 0.009 |  | 0.016 | ± | 0.002 | **<0.001** |
| P-PO_4_^3-^ (mg · L^-1^) | 0.013 | ± | 0.001 |  | 0.019 | ± | 0.004 | 0.170 |
| DIN:P-PO_4_^3-^ | 26.642 | ± | 1.464 |  | 20.677 | ± | 3.532 | 0.126 |

The *P* values are the results of independent *t*-tests comparing physicochemical parameters between reaches. Significant (*P*<0.050) results are highlighted in bold.

^1^Data from [1].

^2^Data from [2].

*High interannual variability as characteristic in a Mediterranean climate.

**References:**

1. Von Schiller D, Martí E, Riera JL, Ribot M, Argerich A, et al. (2008) Inter-annual, Annual, and Seasonal Variation of P and N Retention in a Perennial and an Intermittent Stream. Ecosystems 11: 670–687.

2. Acuña V, Giorgi A, Muñoz I, Sabater F, Sabater S (2007) Meteorological and riparian influences on organic matter dynamics in a forested Mediterranean stream. J North Am Benthol Soc 26: 54–69.
