# Supplementary material for: Consequences of Warming and Resource Quality on the Stoichiometry and Nutrient Cycling of a Stream Shredder
Source: PLoS One. 2015 Mar 4;10(3):e0118520. doi: 10.1371/journal.pone.0118520 (PMC4349742; doi:10.1371/journal.pone.0118520)
Supplement: S2 Table — C, N and P relative egestion rates (least-squares means ± SE) of Echinogammarus (n = 11–15) maintained at 15 and 20°C and fed PERM or INT leaves. (DOCX) [file pone.0118520.s003.docx]

**Table S2. Relative egestion rates of *Echinogammarus*.** C, N and P relative egestion rates (least-squares means ± SE) of *Echinogammarus* (n=11-15) kept at 15 and 20 °C and fed with PERM or INT leaves.

| *Temp (°C)* | *Food quality* | **mg C g^-1^ d^-1^** | | |  | **mg N g^-1^ d^-1^** | | |  | **mg P g^-1^ d^-1^** | | |  |
| --- | --- | --- | --- | --- | --- | --- | --- | --- | --- | --- | --- | --- | --- |
| **15 °C** | **PERM** | 105.26 | ± | 10.97 | ^b^ | 3.36 | ± | 0.36 | ^b^ | 0.111 | ± | 0.013 | ^b^ |
|  | **INT** | 115.11 | ± | 10.77 | ^b^ | 3.96 | ± | 0.36 | ^b^ | 0.128 | ± | 0.013 | ^b^ |
| **20 °C** | **PERM** | 125.42 | ± | 11.12 | ^ab^ | 4.16 | ± | 0.37 | ^b^ | 0.115 | ± | 0.013 | ^b^ |
|  | **INT** | 154.11 | ± | 12.13 | ^a^ | 5.20 | ± | 0.40 | ^a^ | 0.180 | ± | 0.014 | ^a^ |

Different letters indicate significant differences among treatments (*P*<0.050).
